# Home Hazards With Fear of Falling: Findings From the Baseline Study of the Malaysian Elders Longitudinal Research (MELoR)

**DOI:** 10.3389/fpubh.2020.612599

**Published:** 2021-01-12

**Authors:** Muhammad Hibatullah Romli, Lynette Mackenzie, Meryl Lovarini, Lindy Clemson, Maw Pin Tan

**Affiliations:** ^1^Department of Rehabilitation Medicine, Faculty of Medicine and Health Sciences, Universiti Putra Malaysia, Serdang, Malaysia; ^2^Malaysian Research Institute on Ageing (MyAgeing^*TM*^), Universiti Putra Malaysia, Serdang, Malaysia; ^3^Discipline of Occupational Therapy, Sydney School of Health Sciences, Faculty of Medicine and Health, The University of Sydney, Camperdown, NSW, Australia; ^4^Department of Medicine, Faculty of Medicine, University of Malaya, Kuala Lumpur, Malaysia; ^5^Department of Medical Sciences, Faculty of Healthcare and Medical Sciences, Sunway University, Petaling Jaya, Malaysia

**Keywords:** accidental fall, aged, fear of falling (FOF), home safety, fall-related psychological concern

## Abstract

**Background:** Fear of falling (FoF) is a common issue among older people, impacting on psychological health, functional performance and mortality. Many factors associated with fear of falling have been investigated but little is known about the role of home hazards. Home hazards can be due to unsafe environmental and functional features. This study is aims to evaluate the association between home hazards with fear of falling among community-dwelling individuals aged 55 years and over.

**Methods:** Baseline data with 1,489 older individuals from the Malaysian Elders Longitudinal Research (MELoR) study were analyzed. Home visits for interview and observations in the home were conducted with the participants. FoF was established with a single-item question and home hazards with the Home Falls and Accidents Screening Tool (HOME FAST).

**Results:** The majority (76.4%) of older participants experienced FoF. The history of falls was not associated with FoF (*p* = 0.868), but FoF was associated with participants limiting their daily activities (*p* < 0.001). Home hazards were less likely (*p* = 0.023) and functional issues were more likely (*p* < 0.001) to be associated with a high degree of FoF. However, both home hazards domains were not associated with activity restriction due to FoF.

**Conclusions:** Education about home hazards from the perspective of person-environment interaction may encourage home hazards management and reduce FoF which should be evaluated in future studies.

## Introduction

Falls are known as a major issue for older people. Falls are common among community-dwelling older populations (1–4). However, the impact from falls can be catastrophic such as psychological disturbance, injuries (i.e., fractures, pain, oedema), loss of functions (physical, social, activities of daily living), morbidity, and mortality (5). More than 400 risk factors associated with falls have been identified and these can be classified into two major domains; intrinsic and extrinsic (3, 6). While intrinsic factors may include biological, behavior and socioeconomic factors, extrinsic factors are predominantly environmental factors (3, 6). Home hazards are a factor which has received much attention in falls investigations with older people.

Investigating the association between home hazards, falls and risk of falling is difficult and challenging. One review study on the intervention of home hazards management found this to be effective in reducing falls among older people (7). However, observational studies face difficulty proving the association exists. In a review by Letts et al. (8) summarized cross-sectional studies failed to find any significant association, while cohort studies found mixed findings; significant associations were only found in high quality studies. These findings are again replicated in a recent study by Romli et al. (9, 10) that found no significant association between a home hazards score and history of falls. The interaction is complex as either the hazards have been eliminated after the older person had a fall, thus making the prior hazards undetectable, or the hazards appear after a fall due to reduced functionality and daily performance as consequences from the fall. These factors are unstable and there are limitations in investigating these associations through cross-sectional designs.

Fear of falling is considered as an alternative avenue of investigation to understand falls. Fear of falling is the psychological manifestation of falls (11, 12). Previous falls predict the existence of fear of falling in older people and the fear of falling will become a predictor for falls occurrence during the following year (11). In addition, fear of falling has been recognized as a risk factor for falls among older people (13, 14). However, fear of falling is also a factor independent from falls; it is a condition where an older person fears a fall among those who fall and even among those who never fallen (14–16). Fear of falling is worth investigating as more than half of the older population experiences it with potentially serious consequences; psychological impact such as depression, functional and activity restriction, impaired quality of life and increased risk of mortality (16, 17). Using fear of falling as an outcome variable is important as it is persistent over time. Compared to a fall that is only an event at one-point in time, fear of falling is consistent for a period of time and is less likely to be diminished if there is no intervention.

A systematic review found that factors associated with fear of falling include female gender, performance and physical function, the use of a walking aid, history of falls and poor self-rated health. Inconclusive factors were psychological issues (i.e., depression and anxiety) and medication (18). Interventions specifically designed to reduce fear of falling have been found to be effective (19), and these include housing adaptation (20). Identified factors and interventions addressing fear of falling were found to be similar to factors related to falls and interventions for falls prevention (18, 19, 21, 22). This suggests that fall prevention efforts may be transferable to fear of falling. However, home hazards have not been investigated as a factor associated with fear of falling. Only two studies were found and both were from Iran. A study found home hazards to be positively and significantly associated with fear of falling (23). However, the study did not use any standardized instrument specifically to measure home hazards and focused only on three simple items of physical environmental hazard (i.e., present of extra objects, slippery floor or none). Another study found a significant association between higher home hazards and greater fear of falling (24). However, the study utilized the Home Safety Checklist where the instrument was criticized for having poor psychometric properties (9, 10). In addition, the study investigated home hazards as the outcome for fear of falling.

The definition of home hazards has evolved in the past two decades. Home hazards are not merely the presence of danger or deficits in the physical environment but also involve the observation of functional capability of an older person when interacting with the home environment (9, 10, 25, 26). For home hazards, the functional capability of the older person in their home environment might not be determined on the functional independence of older people in general (26). For example, an older person who is independent in activities of daily living and having good balance and mobility may still have a hazard when climbing a narrow flight of stairs or upsetting his or her balance when reaching high objects in the kitchen cabinet. Therefore, there is a need to investigate the role of home hazards on fear of falling among community-dwelling older people in a robust manner.

## Materials and Methods

A cross-sectional study design utilizing data from the first wave of the Malaysian Elders Longitudinal Research (MELoR) project was used in the current study. The first wave data for MELoR serves as the baseline data for a longitudinal cohort study in which the outcomes will inform future government policies and scientific research. Registered voters aged 55 years and over were recruited through simple random sampling stratified by the three major ethnicities (i.e., Malay, Chinese and Indian) in Malaysia and age deciles. Participants were identified through the electoral rolls of three adjacent parliamentary constituencies within the Federal Territory Kuala Lumpur and the Petaling District of the state of Selangor. The recruitment procedures for the project are described elsewhere (27).

### Data Collection

Participants providing informed consent were visited at their home by a trained MELoR researcher using a computer-aided method to administer a set of questionnaires consisting of survey items and validated assessments related to falls. The data were collected through interviews based on the participant's response and via interview-with-observation for the home hazards assessment. The survey captured sociodemographic information, and housing status (i.e., property type, home ownership). The detail of the procedure is reported elsewhere (9, 10).

#### Fear of Falling

Fear of falling was evaluated using a single-item question by asking participants, “Are you afraid of falling?” with a dichotomous answer of “yes” or “no.” If the participants answered “yes” to the initial questions, subsequent questions were then administered, first on the degree of fear by asking, “Do you feel somewhat afraid or very much afraid of falling?” with two answer choices of “somewhat” or “very much,” and second on activity restriction by asking, “Do you limit your activities because you are afraid of falling?” with the selection of answers either “yes” or “no.” The single-item question on fear of falling is widely used and considered a gold standard evaluation due to its simple structure and ease of administration even among people with cognitive impairment (28, 29). It demonstrates acceptable psychometric properties with good discriminant validity compared to the modified Fall Efficacy Scale (*p* < 0.001) and Fall Efficacy Scale–International (FES-I) (*r* = 0.71; *p* < 0.05), as well as good test-retest reliability over a 2-week period (*k* = 0.72) (29).

#### Home Falls and Accidents Screening Tool (HOME FAST)

The HOME FAST is an instrument to screen for home hazards associated with increased risk of falls among older people. Comprising 25 items evaluating seven key areas of hazards on flooring, furniture, lighting, bathroom, storage, stairways and mobility. Physical environment aspects of the HOME FAST comprise 14 items (i.e., item 1,2,3,4,7,8,9,13,14,15,18,19,21, and 23) and functional aspects comprise 11 items (i.e., item 5,6,10,11,12,16,17,20,22,24, and 25) (26). The HOME FAST has been developed considering the influence of the person-environment interaction on the older person's functional performance within the home environment (26). Each item on the HOME FAST is scored “yes” or “no.” With certain items, an additional answer choice of “not applicable” was made available. Only a “no” answer is assigned a 1 mark to indicate the presence of that hazard. The total score ranges from 0 to 25 where higher scores indicate more hazards. The HOME FAST has good face and content validity, strong internal consistency (α = 0.95) good inter-rater (*k* = 0.62–0.85) and excellent test-retest reliability (ICC = 0.77–0.92), and strong predictive validity for falls (OR: 1.016, *p* = 0.006) and responsive (1–2%; OR: 0.984, *p* = 0.02) (1, 2, 9, 10). The HOME FAST has been translated into several languages such as Brazilian-Portuguese, Persian, Mandarin, Malay, and Tamil (9, 10, 30, 31). A brief sample of the HOME FAST is shown in Table 1.

**Table 1:** Example of HOME FAST.

**No**	**Item**	**Y**	**N**	**N/A**
1	Are walkways free of cords and other clutter?			
2	Are floor coverings in good condition?			
3	Are floor surfaces non slip?			
4	Are loose mats securely fixed to the floor?			
5	Can the person get in and out of bed easily and safely?			
6	Can the person get up from their lounge chair easily?			
7	Are all the lights bright enough for the person to see clearly?			
8	Can the person switch a light on easily from their bed?			
9	Are the outside paths, steps and entrances well lit at night?			
10	Is the person able to get on and off the toilet easily and safely?			
11	Is the person able to get in and out of the bath easily and safely?			
12	Is the person able to walk in and out of the shower recess easily and safely?			
13	Is there an accessible/sturdy grab rail/s in the shower or beside the bath?			
14	Are slip resistant mats / strips used in the bath/bathroom/shower recess?			
15	Is the toilet in close proximity to the bedroom?			
16	Can the person easily reach items in the kitchen that are used regularly without climbing bending or upsetting his or her balance?			
17	Can the person carry meals easily and safely from the kitchen to the dining area?			
18	Do the indoor steps/stairs have an accessible/sturdy grab rail extending along the full length of the steps/stairs?			
19	Do the outdoor steps/stairs have an accessible/sturdy grab rail extending along the full length of the steps/stairs?			
20	Can the person easily and safely go up and down the steps/stairs inside or outside the house?			
21	Are the edges of the steps/stairs (both inside and outside the house) easily identified?			
22	Can the person use the entrance door/s safely and easily?			
23	Are paths around the house in good repair, and free of clutter?			
24	Is the person currently wearing well fitting slippers or shoes?			
25	If there are pets – can the person care for them without bending or being at risk of falling over?			

*Y = YES (No hazards)*.

*N = NO (Hazardous)*.

*N/A = Not Applicable*.

### Data Analysis

Missing data in the HOME FAST items (incomplete) were treated by assigning a score of 0 while samples with missing HOME FAST data (no data on all items) were removed. Data on the HOME FAST score were tested for normality and parametric tests were selected. Missing data on the fear of falling question were minimal, treated by using dummy variables and included in the final analysis. The HOME FAST score was the independent variable and was categorized into three types of hazard score: (1) overall score, (2) HOME FAST physical environment score (HF–environment), and (3) HOME FAST functional aspect scores (HF–function). Binary and multinomial logistic regression analyses were conducted depending on the nature of the dependent variable of the fear of falling–two or three categories respectively.

## Results

Initial data were available for 1,489 older participants. The majority of the participants were married (73.6%), living with others (93.9%) and either living in multi-story housing (53.3%) or in owned or spouse-owned houses (75.9%). The majority of older people were afraid of falling (76.4%). Other characteristics were relatively balanced as presented in Table 2. Chi-square analyses found that a history of falls in the previous month was not associated with fear of falling either on falls combined inside and outside the home (*X*_2_ = 0.028; *p* = 0.868) or falls inside home only (*X*_2_ = 1.434; *p* = 0.231). However, the presence of fear of falling was strongly associated with the perception of limitations of daily activities (*X*_2_ = 1,351.97; *p* < 0.001). The mean number of hazards in the participants' home as recorded by the HOME FAST was 5.23 (95%CI = 5.10–5.37). Detailed types of hazards as measured by the HOME FAST can be referred to in another publication (9, 10).

**Table 2:** Demographic characteristics of the participants.

**Demographic**		***N* (total valid)**	***n* (%)**
Age [mean (95%CI)]		1,453	68.71 (68.32–69.09)
Gender	Male	1,486	649 (43.7)
	Female		837 (56.3)
Ethnicity	Malay	1,483	507 (34.2)
	Chinese		480 (32.4)
	Indian		488 (32.9)
	Others		8 (0.5)
Religion	Islam	1,469	518 (35.3)
	Christian		272 (18.5)
	Buddhist		255 (17.4)
	Hindu		377 (25.7)
	Others		47 (3.2)
Marital status	Single/never married	1,479	83 (5.6)
	Married		1,089 (73.6)
	Divorced/separated/widowed		307 (20.8)
Household	Alone	1,484	91 (6.1)
	With others		1,393 (93.9)
Education	None or primary	1,481	421 (28.4)
	Secondary		619 (41.8)
	Tertiary		441 (29.8)
Type of property	Landed single-story house	147	434 (29.4)
	Landed multi-story house		788 (53.3)
	Apartment-like		217 (14.7)
	Traditional house		39 (2.6)
Home ownership	Own or spouse owned	1,480	1,123 (75.9)
	Not own		357 (24.1)
Years living in their home, years [mean (95%CI)]		1,453	29.95 (29.25–30.64)
Falls in past 12 months	No	1,464	1,132 (77.3)
	Yes		332 (22.7)
	Falls not at home	332	150 (45.2)
	Falls at home		182 (54.8)
Fear of falling	No	1,459	345 (23.6)
	Yes		1,114 (76.4)
	Somewhat	1,109	506 (45.6)
	Very much		603 (54.4)
	Not limit activities	1,104	590 (53.4)
	Limit activities		514 (46.6)
HOME FAST [mean (95%CI)]		1,489	5.23 (95%CI = 5.10–5.37)

### Association Between Home Hazards and Fear of Falling

Due to missing data on the HOME FAST, only data from 1,374 participants were included in the final analysis. Binary logistic regression analysis on the total score of the HOME FAST was found to have no significant association with fear of falling [Exp(β) = 1.010; *p* = 0.694]. Further analysis was conducted through binary logistic regression according to the HOME FAST subscales which found that fear of falling was associated with lower HF-environmental scores [Exp(β) = 0.919; *p* = 0.029] and higher of HF-functional scores [Exp(β) = 1.193; *p* = 0.003].

Additional findings from the multinomial logistic analysis revealed that home hazard scores were not associated with fear of falling among older participants who were ‘somewhat' fearful compared to those with no fear either on HF-environment (*p* = 0.566) or HF-function (*p* = 0.963). However, the rating of ‘very much' fearful of falling was associated with reduced HF-environment (*p* = 0.023) score and but increased HF-function score (*p* < 0.001) when compared to individuals with no fear (see Table 3).

**Table 3:** Association between fear of falling and home hazards using multinomial logistic regression.

**HF subscale**	**Fear of falling**	**HF score mean (95%CI)**	**Exp (B)**	**Wald**	**S.E**.	***P*-value**
Environment	Very much^†^	3.77 (3.61–3.92)	0.909	5.139	0.042	0.023^*^
	Somewhat^†^	3.78 (3.63–3.93)	0.976	0.330	0.043	0.566
	No fear	3.86 (3.66–4.06)	*reference*
Function	Very much^†^	1.63 (1.51–1.75)	1.282	16.256	0.062	< 0.001^**^
	Somewhat^†^	1.30 (1.20–1.40)	1.003	0.002	0.067	0.963
	No fear	1.31 (1.19–1.43)	*reference*

† *Compared with no fear*.

* *p ≤ 0.05*,

** *p ≤ 0.01*.

Among those who were fearful of falling (*n* = 1,039), binary logistic regression yielded no significant association of home hazards with whether or not the participants limited or did not limit their daily activities due to fear of falling on both HOME FAST subscales; HF-environment (*p* = 0.205) or HF-function (*p* = 0.105).

## Discussion

This study indicates those with a high level of fear of falling were less likely to have environmental home hazards but were more likely to have functional home hazards compared to individuals with no fear of falling. When all home hazards are evaluated together we found no association with presence fear of falling or level of fear of falling. Environmental home hazards may have been reduced when individuals develop fear of falling; these individuals may be more vigilant (17) and hence may have taken active steps to reduce environmental hazards. Conversely, the presence of functional home hazards may have precipitated fear of falling as these individuals with functional home hazards are more likely to experience challenges in maintaining stability while performing daily tasks and are more likely to perceive the threat of falls. Previous studies had, however, found an association between home hazards and fear of falling (23, 24). However, the method of identifying home hazards was not described (23). The presence of home hazards also did not involve the use of a checklist through direct observation (24). Therefore, this study where the home hazards were evaluated via observation and interview provides stronger confidence in the outcome.

The fear avoidance model indicates that an individual may rectify any problem or avoid an activity that may cause a fall (32). Fear of falling is influenced by balance problems and falls, and cognitive issues–particularly attention and processing of sensory information–which may elicit the fear (32). Older people who are afraid may become meticulous in subconsciously identifying any hazards in their environment. The older person or family members may have rectified any physical hazards available. This makes the environment safer and reduces the perception of risk of falling (33). Meanwhile, older people who are not afraid may not be attentive to any physical hazards present and therefore do not eliminate or modify them. If the fear of falling is low there may not be sufficient motivation to correct the hazards (34). However, the fear-avoidance model may also negatively affect an older people doing functional tasks which may then cause the older person to be less competent in functional performance to overcome the hazards. Any effort to eliminate hazards might also be limited due to lack of knowledge and limited resources (e.g., financial, material) as these actions are self-driven. Identification and management of home hazards may be limited without appropriate health professional input (i.e., occupational therapist) and only simple modifications may be done due to cost (33, 35).

A higher risk of home hazards was found to not restrict the older participants from doing their activities of daily living. One reason is the number of hazards in this study is considered low. A study in Australia considered a cut-off point of 9 and above while a study in Asia considered the cut-off point at 6 and above as having a high risk level of home hazards (36, 37). The HOME FAST is a screening tool which is brief and aims to identify individuals at risk of having significant home hazards. However, the limited number of items may not be able to identify hazards comprehensively compared to other assessment tools (9, 10, 38). This has reduced the ability of the HOME FAST to detect older people who are truly at risk of having home hazards that limit daily function. Therefore, future study should utilize comprehensive home hazards assessment such as Westmead Home Safety Assessment (WeHSA) for a thorough evaluation and detailed outcome.

Although reduced functional performance *per se* is considered an established risk factor for falls and fear of falling among older people, the definition of functional performance inherent in the assessment of home hazards is different. Hazardous functioning as part of a home hazard happens only when an older person is performing an unsafe activity due to an unsafe environment or the presence of hazardous environments on the risk of falling, while another assessment may consider that independence in functions are intact. For example, if the older person needs to stand on tip-toes when reaching high items in the kitchen cabinet, this may be considered a hazardous activity in terms of falls risk even though the person may have good balance and mobility skills. Our study is unique in that we have identified that the interaction between the older person and the home environment is related to fear of falling; although the participants in this study were relatively independent in basic and instrumental activities of daily living as previously reported in Romli et al. (9, 10). This finding supports the hypothesis by Iwarsson et al. (25) where the interaction between the person and their hazardous environment increases the risk of falling. This indicates that a safer home environment is important to facilitate the prevention of falls.

The limitation of this study is that some of the home hazards might be unsuccessfully identified by the MELoR researchers who are not occupational therapists as they have limited knowledge on human body structure and functions, and occupational science. The concept of person-environment fit is complex. Dynamic variability on how older adults navigate their home environment in ways that either reduce or increase their risk of falls is difficult to capture (39). The functional aspects may involve underlying contributions such as cognitive, vision and mobility problems that may be difficult for non-health or non-occupational therapy trained individuals to interpret when conducting the assessment (1, 2, 31). Another limitation is the cross-sectional design where any causal-and-effect relationship is unable to be determined (40) however prospective data is not yet available. There is no study that thoroughly investigated fear of falling in Malaysia and this MELoR project is the pioneer for such research while older Malaysians do not recognize fear of falling and its impact (41). Moreover, stroke is a major issue with older people and combined with falls has made the issue significant (42). Stroke survivors have been reported with a higher level of fear of falling compared to the general population in Malaysia (43). Hence, with the absence of studies in Malaysia on fear of falling, this study is valuable.

Management of the physical environment is important to reduce environmental hazards and functional hazards. However, this action is under-appreciated by older people as they view that home modification has few benefits and falls usually happen due to their own incapacity (41). Older people mostly had high fear of falling when performing activities at home (13). Fear of falling is common among community-dwelling older people and is independent of whether or not a person experiences a fall. Fear of falling is influenced by an older person's perception of their own physical or functional capability to mediate their surroundings (13). Learning from other older people who fell and the consequences from the fall may elevate the fear on falls among older people (44). Fear of falls can reduce the functional capacity of older people because they may limit their daily activities as a consequence of the fear. However, well-managed home hazards can reduce falls and fear of falling (7, 20). Public education on home hazards conducted from the person-environment interaction perspective should, therefore, be explored as a potential solution to reducing fear of falling among older adults. As fear of falling is not associated with falls, future studies should also consider evaluating the relationship between fear of falling and quality of life and health outcomes in this population.

## Data Availability Statement

The data analyzed in this study is subject to the following licenses/restrictions: The dataset can be requested to the AGELESS Longitudinal Study of Ageing. Requests to access these datasets should be directed to ageless@um.edu.my.

## Ethics Statement

The studies involving human participants were reviewed and approved by University of Malaya Research Ethics Committee (MEC Ref No: 943.6). The patients/participants provided their written informed consent to participate in this study.

## Author Contributions

MHR has major contribution on initiating the original idea and writing the manuscript conducting the systematic searching. MHR, LM, ML, LC, and MPT have equal contribution on disseminating, critical analyzing, and synthesizing the findings. MPT was one of the principal investigators for MELoR project back then and currently the head of principal investigator for AGELESS Longitudinal Study for Ageing. All authors approved the final version of the manuscript.

## Conflict of Interest

The authors declare that the research was conducted in the absence of any commercial or financial relationships that could be construed as a potential conflict of interest.
